# Supplementary material for: Arachidonoylcholine and Other Unsaturated Long-Chain Acylcholines Are Endogenous Modulators of the Acetylcholine Signaling System
Source: Biomolecules. 2020 Feb 12;10(2):283. doi: 10.3390/biom10020283 (PMC7072677; doi:10.3390/biom10020283)
Supplement: Supplementary file 1 [file biomolecules-10-00283-s001.pdf]

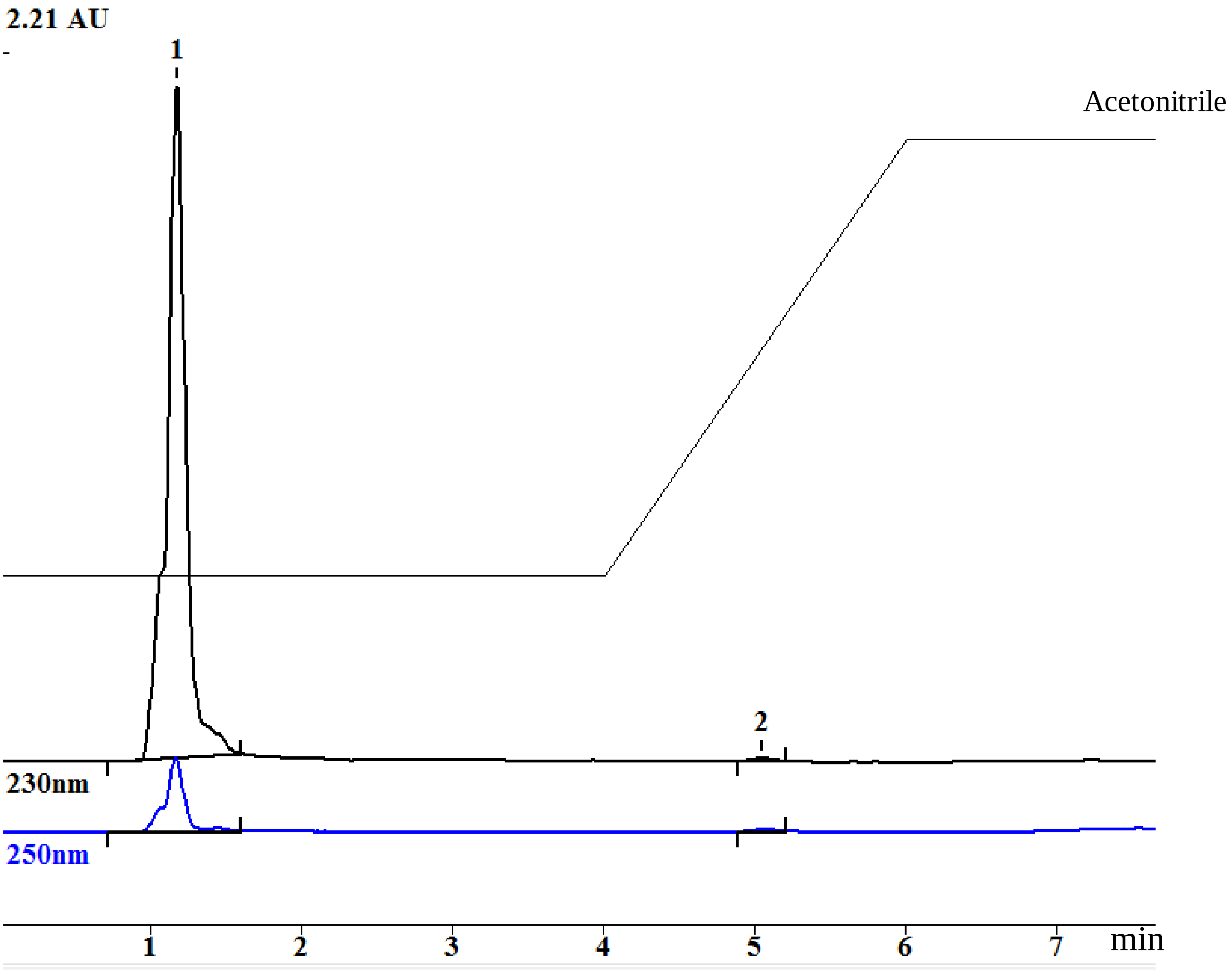

Figure S1. Purity check of AA-CHOL. HPLC data. Column ProntoSil 120-5-C18 AQ; gradient elution with 0.1% trifluoroacetic acid in acetonitrile/H_2O. Peak 1 is the target compound. Calculated compound purity is 99.4%.

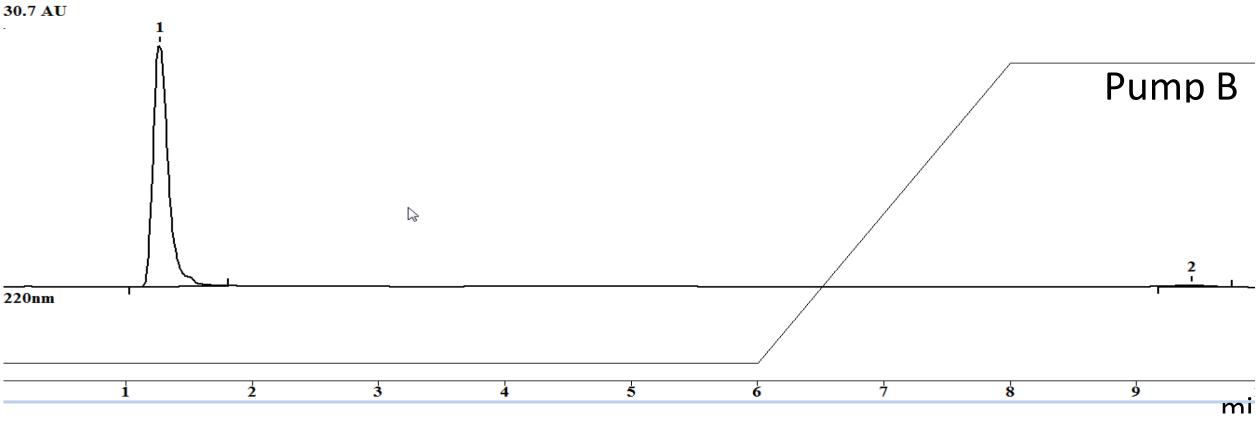

Figure S2. Purity check of Ol-CHOL. HPLC data. Column ProntoSil 120-5-C18 AQ; gradient elution with 0.1% trifluoroacetic acid in acetonitrile/H_2O. Calculated compound purity is 98.7%.

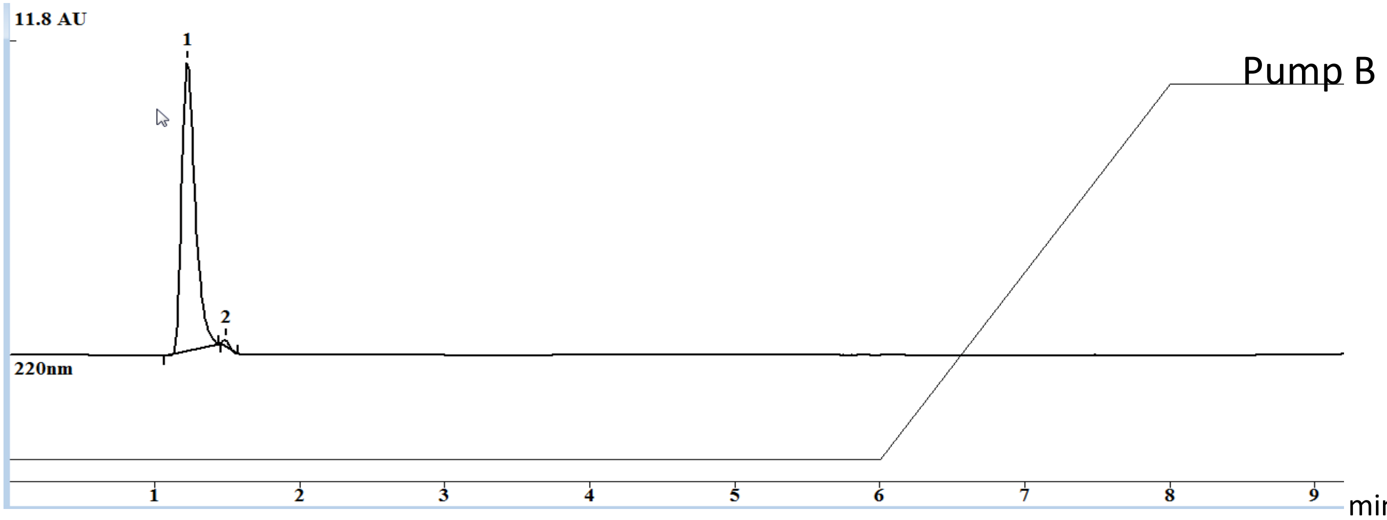

Figure S3. Purity check of Ln-CHOL. HPLC data. Column ProntoSil 120-5-C18 AQ; gradient elution with 0.1% trifluoroacetic acid in acetonitrile/H_2O. Calculated compound purity is 99.4%.

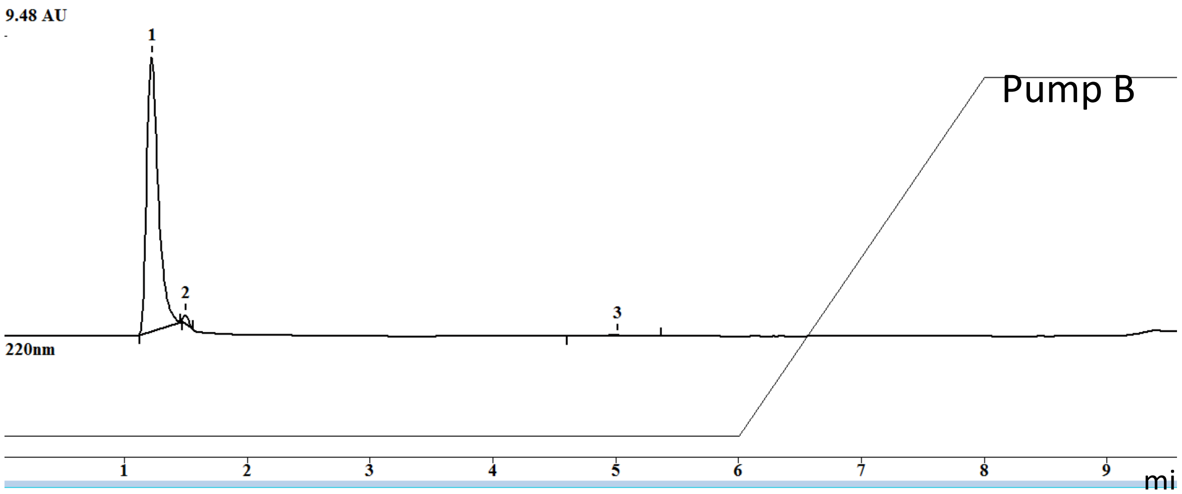

Figure S4. Purity check of DHA-CHOL. HPLC data. Column ProntoSil 120-5-C18 AQ; gradient elution with 0.1% trifluoroacetic acid in acetonitrile/H_2O. Calculated compound purity is 97.9%.

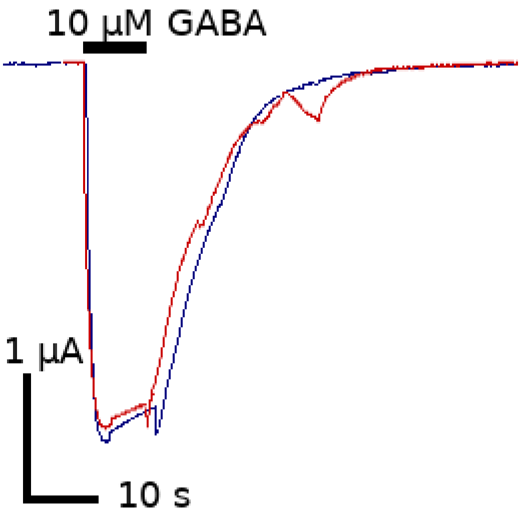

Figure S5. GABA-evoked currents in Xenopus oocyte expressing murine α1β3γ2 GABAA receptor: control (blue line) and under 10 µM AA-Chol treatment. AA-Chol was pre-applied 5 min before GABA and was added to GABA solution during the application. Current amplitude does not show significant difference from the control (99 + 6%, n=3, $p<0.05$, Student's t-test).

© 2019 by the authors. Submitted for possible open access publication under the terms and conditions of the Creative Commons Attribution (CC BY) license (http://creativecommons.org/licenses/by/4.0/).
